# A novel genomic instability-derived lncRNA signature to predict prognosis and immune characteristics of pancreatic ductal adenocarcinoma

**DOI:** 10.3389/fimmu.2022.970588

**Published:** 2022-09-15

**Authors:** Huijie Yang, Weiwen Zhang, Jin Ding, Jingyi Hu, Yi Sun, Weijun Peng, Yi Chu, Lingxiang Xie, Zubing Mei, Zhuo Shao, Yang Xiao

**Affiliations:** ^1^ National Clinical Research Center for Metabolic Diseases, Key Laboratory of Diabetes Immunology, Ministry of Education, and Department of Metabolism and Endocrinology, The Second Xiangya Hospital, Central South University, Changsha, China; ^2^ Department of Pathology, The Second Xiangya Hospital, Central South University, Changsha, China; ^3^ Department of Integrated Traditional Chinese and Western Medicine, The Second Xiangya Hospital, Central South University, Changsha, China; ^4^ Department of Gastroenterology, The Second Xiangya Hospital, Central South University, Changsha, China; ^5^ Department of Anorectal Surgery, Shuguang Hospital Affiliated to Shanghai University of Traditional Chinese Medicine, Shanghai, China; ^6^ Anorectal Disease Institute of Shuguang Hospital, Shanghai, China; ^7^ Department of General Surgery, Changhai Hospital, Naval Medical University, Shanghai, China

**Keywords:** long non-coding RNAs, genome instability, tumor microenvironment, pancreatic ductal adenocarcinoma, epithelial-mesenchymal transition

## Abstract

**Background:**

Pancreatic ductal adenocarcinoma (PDAC) is a highly aggressive malignant tumor of the digestive system. Its grim prognosis is mainly attributed to the lack of means for early diagnosis and poor response to treatments. Genomic instability is shown to be an important cancer feature and prognostic factor, and its pattern and extent may be associated with poor treatment outcomes in PDAC. Recently, it has been reported that long non-coding RNAs (lncRNAs) play a key role in maintaining genomic instability. However, the identification and clinical significance of genomic instability-related lncRNAs in PDAC have not been fully elucidated.

**Methods:**

Genomic instability-derived lncRNA signature (GILncSig) was constructed based on the results of multiple regression analysis combined with genomic instability-associated lncRNAs and its predictive power was verified by the Kaplan-Meier method. And real-time quantitative polymerase chain reaction (qRT-PCR) was used for simple validation in human cancers and their adjacent non-cancerous tissues. In addition, the correlation between GILncSig and tumor microenvironment (TME) and epithelial-mesenchymal transition (EMT) was investigated by Pearson correlation analysis.

**Results:**

The computational framework identified 206 lncRNAs associated with genomic instability in PDAC and was subsequently used to construct a genome instability-derived five lncRNA-based gene signature. Afterwards, we successfully validated its prognostic capacity in The Cancer Genome Atlas (TCGA) cohort. In addition, *via* careful examination of the transcriptome expression profile of PDAC patients, we discovered that GILncSig is associated with EMT and an adaptive immunity deficient immune profile within TME.

**Conclusions:**

Our study established a genomic instability-associated lncRNAs-derived model (GILncSig) for prognosis prediction in patients with PDAC, and revealed the potential functional regulatory role of GILncSig.

## Highlights

We established a mutational hypothesis-derived computational framework for identifying genomic instability-associated lncRNAs in PDAC and constructed lncRNA signatures to better predict the prognosis of PDAC patients.

In addition, we investigated the immune profile characteristics and potential functional regulatory effects associated with lncRNA signatures, and we found that GILncSig is associated with EMT as well as adaptive immune deficiency immune profiles within the TME.

Our findings may improve prognostic prediction methods for PDAC and provide potential guidance for precise immunotherapy in the future.

## 1 Introduction

Pancreatic ductal adenocarcinoma is one of the aggressive solid malignancies, and the rising incidence of PDAC is expected to be the second leading cause of cancer-related mortality by 2030 (1, 2). However, only 10% to 20% of pancreatic cancer patients have the chance of surgery as most patients have distant metastasis of the lesion at the time of diagnosis (3). Moreover, even for patients with the chance of surgery, they still possessed a rather low 5-year survival rate and over 80% postoperative recurrence rate (4). Despite recent advances in pancreatic cancer research, there has been no significant reduction in overall mortality and morbidity (5), because of the lack of specific symptoms and reliable biomarkers for early diagnosis, as well as poor response to treatment due to tumor dissemination. Therefore, there is an urgent need to develop new and effective strategies that can predict prognosis and improve therapeutic targeting to achieve personalized treatment.

Genomic instability is shown to be an important cancer characteristic as well as a prognostic factor, and the pattern and degree of which is associated with tumor progression and recurrence (6, 7). Although the specific molecular mechanisms affecting genomic instability are not fully understood yet. Recently, long non-coding RNAs, a group of non-coding RNAs with more than 200 nucleotides in length (8, 9), are considered to have the potential to quantitatively measure genomic instability (10–16). Interestingly, quite a few previous studies have reported a variety of lncRNAs that may contribute to the carcinogenesis and development of PDAC (17–19). Therefore, we believe that lncRNAs may represent a new class of PDAC biomarkers and therapeutic targets. Likewise, genomic-instability related lncRNAs were successfully used to build prognostic models for other types of cancer, including breast cancer, gastric cancer, and glioblastoma (20–22).

Besides, it is widely believed that studies targeting the crosstalk between tumor cells and the TME will shed light on the novel treatment measures for pancreatic cancer. Immune checkpoint inhibitors have been reported to show durable clinical benefits in many malignancies (23). However, we found that the effect of this class of drugs was not satisfactory in PDAC, which may be ascribed to the distinct TME profile. Cancer often creates a favorable TME for its successful growth by disrupting the immune, vascular, and connective tissue components of the stroma that counter the physiological responses to damage. Among them, the intensive interstitial and highly immunosuppressive environment is a special weapon for PDAC (24–26). At the same time, the degree of T-cell infiltration in PDAC patients correlates with disease progression, and patients with a higher level of T-cell infiltration are generally more sensitive to immunotherapy (27, 28).

In this study, we employed bioinformatics and statistics methods, combined with the lncRNA expression profile of tumor genomes, the somatic mutation profile of tumor genomes, and the clinical features of PDCA patients to establish a genomic-instability associated lncRNA-derived signature called GILncSig. The GILncSig risk score was calculated as a surrogate tool for assessing the likelihood of survival in patients with PDCA, and its prognostic value was further validated by survival analysis. Moreover, to understand the concomitant functional regulatory effects of GILncSig on the transcriptomic expression profiles, we conducted graph-based clustering analysis, differential expression analysis and functional enrichment analysis to in-depth analyze transcriptome expression characteristics associated with GILncSig, revealing that GILncSig is related to EMT. In addition, we investigated the lncRNA signature associated immune profile characteristics and potential functional regulatory effects. We believe that our findings may improve the prognostic prediction method of PDAC and provide potential guidance for future precise immunotherapy.

## 2 Materials and methods

### 2.1 Data collection

RNA-seq expression data, clinical features, clinicopathological characteristics, survival information, and somatic mutation information of patients with pancreatic ductal adenocarcinoma were collected from TCGA database (https://portal.gdc.cancer.gov/). LncRNA expression data were downloaded from the TANRIC database (http://bioinformatics.mdanderson.org/main/TANRIC: Overview, version 1.0.6). Due to the missing values in the follow-up dataset, 7 patients from the TCGA cohort were excluded, and the remaining 171 samples were retained for further study. A flow chart of the inclusion and exclusion criteria for patient data is presented in **Supplementary Figure 1**. The patients with PDAC used in this study were randomly divided into the following two patient sets after matching for gender, age, and tumor stage: training set (n = 87) and test set (n = 84), with no significant differences in clinical features between these two sets. The training set was used to identify prognostic lncRNA signature and build prognostic risk model, while the testing set was used to independently validate the performance of the prognostic risk model.

### 2.2 Identification of genome instability-associated lncRNAs

As described in the previous study, a mutator hypothesis-derived computational frame combining lncRNA expression profiles and somatic mutation profiles in a tumor genome was used to identify genome instability-associated lncRNAs (29). In brief, the cumulative number of somatic mutations was first calculated for each pancreatic cancer patient and sorted in descending order. Next, the first 25% and last 25% of patients were defined as genomic instability (GU) and genomic stability (GS) sample groups, respectively. Finally, the expression profiles of lncRNAs of the GU and GS groups were compared using the significance analysis of microarrays (SAM) method, and the differentially expressed lncRNAs between these two groups (fold change > 1.5 or<0.67 and false discovery rate (FDR) adjusted P< 0.05) were defined as genome instability-associated lncRNAs (29).

### 2.3 Functional enrichment analysis

To understand the potential functional regulation exerted by the identified genome instability-associated lncRNAs, an exploratory graph-based clustering analysis using the Louvain clustering algorithm was performed on the entire TCGA dataset, identifying three distinct clusters in all pancreatic ductal adenocarcinoma patients. Functional enrichment analysis of the extracted top 50 differentially expressed genes and top 30 differentially expressed transcription factors (TFs) from each cluster was conducted using the Metascape webtool (www.metascape.org) to determine significantly enriched Gene Ontology (GO) terms and Kyoto Encyclopedia of Genes and Genomes (KEGG) pathways (30). As a result, we found that cluster 1 was implicated in EMT.

### 2.4 Statistical analysis

We used univariate and multivariate Cox proportional hazard regression analysis to evaluate the association between the expression level of genome instability-associated lncRNA and overall survival. According to the coefficients from the multivariate regression analysis and the expression levels of prognostic genome instability-associated lncRNAs, a genome instability-derived lncRNA signature for outcome prediction was constructed as follows:

GILncSig = $\sum_{i=1}^{n}coef(IncRNAi)\;*\;\exp r(IncRNAi)$ , (i=1,2,3…n)

In this equation, GILncSig, the established prognostic risk score for patients with pancreatic ductal adenocarcinoma, is calculated by adding up the product of the coefficient of each lncRNA derived from the multivariate regression analysis and its expression level. The median score of the patients in the training set was used as the risk cutoff value to classify patients into either a high-risk group with high GILncSig or a low-risk group with low GILncSig.

Median survival and survival rates were calculated for each prognostic risk group using the Kaplan-Meier method, and the log-rank test was used to assess the survival difference between the high-risk and low-risk groups at the 5% significance level. The independence of GILncSig from other key clinical factors was clarified by multivariate Cox regression and stratification analysis. The performance of GILncSig was also assessed by receiver operating characteristic (ROC) curves. Unsupervised hierarchical clustering analysis for the identification of GU-like and GS-like groups was performed using Euclidean distance and Ward’s linkage method, whereas unsupervised hierarchical clustering analysis for the investigation of the potential functional regulatory role of GILncSig was performed using Louvain clustering algorithm and graph-based clustering method. The association between GILncSig risk groups and cluster 1 was consolidated *via* Pearson correlation analysis between GILncSig risk score and EMT signature score. All statistical analyses were performed using R-version 3.6.

### 2.5 Gene set enrichment analysis

Gene set enrichment analysis was carried out using two EMT signatures constructed from previously reported literature (31, 32). Enriched-ness of gene expression in each gene set of patients was defined by a signature score calculated through R VISION package.

### 2.6 Estimated the immune profile of tumor microenvironment

To better understand the GILncSig-related immune landscape, we used the CIBERSORT algorithm (https://cibersort.stanford.edu/index.php) combined with LM22 to estimate the abundances of immune cell subsets within the TME in each patient, as designed in the previous study (33). An empirical P-value for the deconvolution using Monte Carlo sampling was thereby produced, and cases with a resulting P-value< 0.05 were available for further analysis. We then estimated the Immune Score, namely the ratio of immune matrix components in the TME of each sample, by the ESTIMATE algorithm (34). The higher the Immune Score, the larger the ratio of the immune components in TME. Furthermore, the correlation between GILncSig risk scores and Immune Score was validated by Pearson correlation analysis.

### 2.7 Tissue specimens

Five formalin-fixed paraffin-embedded pancreatic ductal adenocarcinoma tumor specimens and their adjacent tissues were obtained from the pathology department of the hospital, and qRT-PCR was performed on these tissues. These patients met the following inclusion criteria: (1) Adult patients aged ≥ 18 years and ≤ 75 years, histologically (non-cytologically) diagnosed with PDAC; (2) Patients with stage I-III according to the 8th edition of the American Joint Committee on Cancer (AJCC) classification; (3) Patients with a life expectancy ≥3 months. The exclusion criteria were: (1) Patients diagnosed with other types of pancreatic malignant tumor or malignant tumor(s) of other tissues; (2) Patients with other severe concomitant disease or disorder such as heart, liver, or renal failure; (3) Patients having no adjacent non-cancerous tissue in the paraffin-embedded pancreatic tissue. This study was approved by the local Ethics Committee (Second Xiangya Hospital Ethics Committee) (approved no. 2020-465). The requirement for written informed consent was waived for the retrospective tissue samples included in the pancreatic ductal adenocarcinoma tumor specimens.

### 2.8 RNA isolation and qRT-PCR analysis

Total RNA was prepared using The ReliaPrep™ FFPE Total RNA Miniprep System (Promega) according to the manufacturer’s instructions. The concentration of the total RNA was detected by NanoDrop 2000 (Thermo Scientific™). Total RNA (1000 ng) was reverse transcribed into cDNA using RevertAid First Strand cDNA Synthesis Kit (Thermo Scientific™). The relative expression of target genes to the housekeeping gene GAPDH was determined by qRT-PCR using GoTaq^®^ qPCR Master Mix (Promega). All primer sequences used in this study were listed in **Supplementary Table 1**. Analysis between the two groups was performed by an unpaired t-test; P< 0.05 was considered statistically significant.

## 3 Results

### 3.1 Identification of genomic instability-related lncRNAs in pancreatic cancer patients

#### 3.1.1 Identification of genomic instability-related lncRNAs

The flowchart of the study is described in **Supplementary Figure 2**. To identify lncRNAs associated with genomic instability, we calculated the cumulative number of somatic mutations per patient and sorted them in ascending order. The top 25% (n = 40) and the last 25% (n = 43) of patients were assigned to GS-like and GU-like groups based on the cumulative number of somatic mutations. Next, a total number of 206 differentially expressed lncRNAs (labeled as DE lncRNAs) between GU-like and GS-like groups were identified based on the comparison of their lncRNA expression profiles (with the absolute value of logFC greater than 1 and FDR-adjusted P-value less than 0.05). Among them, 95 lncRNAs were upregulated and 111 lncRNAs were downregulated in the GU-like group.

#### 3.1.2 Clustering analysis for patient classification

**Figure 1:**
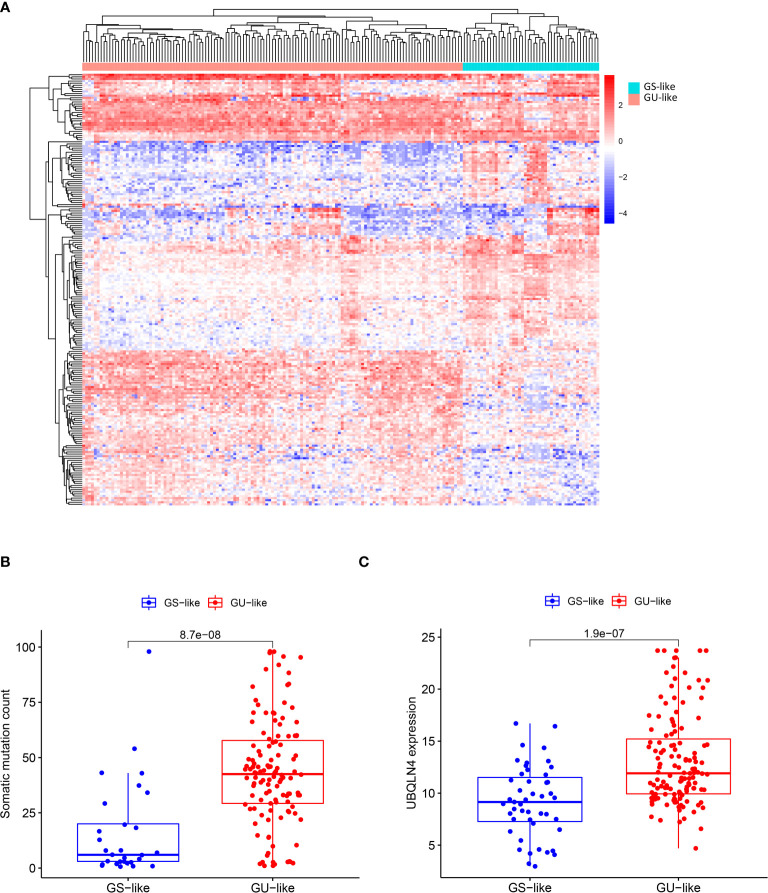
Identification of genomic instability-related lncRNAs in pancreatic cancer patients. **(A)** Unsupervised clustering of 178 patients with Pancreatic ductal adenocarcinoma based on expression patterns of 206 candidate genomic instability-associated lncRNAs. The red cluster on the left is GU-like group and the blue cluster on the right is GS-like group. **(B)** Box plot of somatic mutations in the GU-like group and GS-like group. Somatic cumulative mutations were significantly higher in the GU-like group than in the GS-like group. **(C)** Box plot of UQLN4 expression levels in the GU-like group and GS-like group. The expression level of UBLNQ4 was significantly higher in the GU-like group than in the GS-like group. Horizontal line: median value. Statistical analysis was performed using the Mann-Whitney U test.

To classify all 178 TCGA patients into either GU-like or GS-like groups, unsupervised hierarchical clustering analysis was performed using 206 DE lncRNAs. As shown in **Figure 1A**, all 178 samples were clustered into two groups based on the expression levels of the 206 DE lncRNAs. The GU-like group has significantly higher cumulative somatic mutation counts compared with the GS-like group (P< 0.001, Mann–Whitney U test; **Figure 1B**). Comparison of the expression level of UBQLN4 gene, a newly identified biomarker for genomic instability, reveals that the GU-like group has a significantly higher expression level of UBQLN4 compared with the GS-like group (P< 0.001, Mann–Whitney U test; **Figure 1C**).

### 3.2 Establishment of genomic instability-derived lncRNA signature and outcome prediction

#### 3.2.1 Screening of prognostic-related lncRNAs using Cox proportional hazard regression analysis in the training set

To explore the potential prognostic values of 206 DE lncRNAs, 7 pancreatic cancer patients from the TCGA cohort were excluded due to missing values in their follow-up dataset. The remaining 171 pancreatic cancer patients were randomly split into 2 sets: training set (n = 87) and testing set (n = 84). Statistical comparison of key clinical features between the patients within the training set and the testing set revealed that no significant differences exist between patients in these 2 sets (**Table 1**). The training set will be used for the following procedures. To screen for lncRNAs that can be used as prognostic factors, univariate Cox proportional hazard regression analysis was first performed to determine if the expression level of each DE lncRNA is significantly associated with the prognosis, i.e, the overall survival (OS) of pancreatic cancer patients. As a result, 22 DE lncRNAs were identified (P< 0.05, **Table 2**). Next, these 22 candidate DE lncRNAs were subjected to multivariate Cox proportional hazards regression analysis with common clinical features such as age, gender, and tumor grade to further screen lncRNAs with prognostic ability independent of other lncRNAs. Subsequently, five lncRNAs (TM4SF1-AS1, CASC8, PRDM16-DT, LINC00996, AP000892.3, labeled siglncRNAs) were identified (P< 0.1, **Table 3**) as independent prognostic factors.

**Table 1:** Clinical information for three TCGA patients sets in this study.

Covariates	Type	TCGA set	Training set	Testing set	P value
Age	<=65	90 (52.63%)	50 (57.47%)	40 (47.62%)	0.2556
	>65	81 (47.37%)	37 (42.53%)	44 (52.38%)	
Gender	FEMALE	78 (45.61%)	40 (45.98%)	38 (45.24%)	1
	MALE	93 (54.39%)	47 (54.02%)	46 (54.76%)	
Grade	G1-2	120 (70.18%)	63 (72.41%)	57 (67.86%)	0.467
	G3-4	49 (28.65%)	22 (25.29%)	27 (32.14%)	
	Unknown	2 (1.17%)	2 (2.3%)	0 (0%)	
Stage	Stage I-II	161 (94.15%)	82 (94.25%)	79 (94.05%)	0.9743
	Stage III-IV	7 (4.09%)	3 (3.45%)	4 (4.76%)	
	unknown	3 (1.75%)	2 (2.3%)	1 (1.19%)	
T	T1-2	28 (16.37%)	14 (16.09%)	14 (16.67%)	1
	T3-4	141 (82.46%)	72 (82.76%)	69 (82.14%)	
	Unknown	2 (1.17%)	1 (1.15%)	1 (1.19%)	
M	M0	77 (45.03%)	39 (44.83%)	38 (45.24%)	0.662
	M1	4 (2.34%)	3 (3.45%)	1 (1.19%)	
	Unknown	90 (52.63%)	45 (51.72%)	45 (53.57%)	
N	N0	47 (27.49%)	19 (21.84%)	28 (33.33%)	0.1155
	N1-3	119 (69.59%)	66 (75.86%)	53 (63.1%)	
	Unknown	5 (2.92%)	2 (2.3%)	3 (3.57%)	

**Table 2:** Univariate Cox proportional hazard regression analysis identified 22 DE lncRNAs that are significantly associated with the overall survival of pancreatic cancer patients.

lncRNA	Hazard Ratio	P Value
PRDM16-DT	0.72247472	0.01877915
BX640514.2	1.31449287	0.00069123
AC008969.1	0.29543606	0.03292011
LINC00996	0.30959956	0.04539756
AL121929.3	0.61423882	0.0292435
AC120049.1	0.24663223	0.01804794
LINC02716	0.17449104	0.04206901
LINC02577	1.35591727	0.04757554
AC132938.2	0.23067713	0.0080087
SOCS2-AS1	0.22994143	0.02242125
TM4SF1-AS1	1.45664584	0.00014889
AL355803.1	0.39480085	0.02972008
AP000892.3	0.35868049	0.01728944
LINC01133	1.01990993	0.00016229
AP000757.2	0.78413922	0.02385144
LINC02041	1.13331718	0.00108853
AC087752.3	0.28291938	0.04381474
AL359504.1	0.1520814	0.00430568
AC104695.4	1.12588936	0.04270116
CASC8	1.2432148	0.00063661
SH3PXD2A-AS1	1.1224769	0.03158918
AC015911.3	0.3222307	0.03580903

**Table 3:** Multivariate Cox proportional hazard regression analysis of the 22 prognosis-related DE lncRNAs further narrowed down to 5 DE lncRNAs that are independently associated with the overall survival of pancreatic cancer patients.

lncRNA	Coefficient	Hazard ratio	P value
PRDM16-DT	-0.2782435	0.75711245	0.05078589
LINC00996	-1.1024105	0.33206967	0.09393344
TM4SF1-AS1	0.29167955	1.33867397	0.00544925
AP000892.3	-1.042062	0.35272661	0.02984887
CASC8	0.16646916	1.18112711	0.02519788

#### 3.2.2 Construction of GILncSig and outcome prediction for the training set, testing set, and combined TCGA set

**Figure 2:**
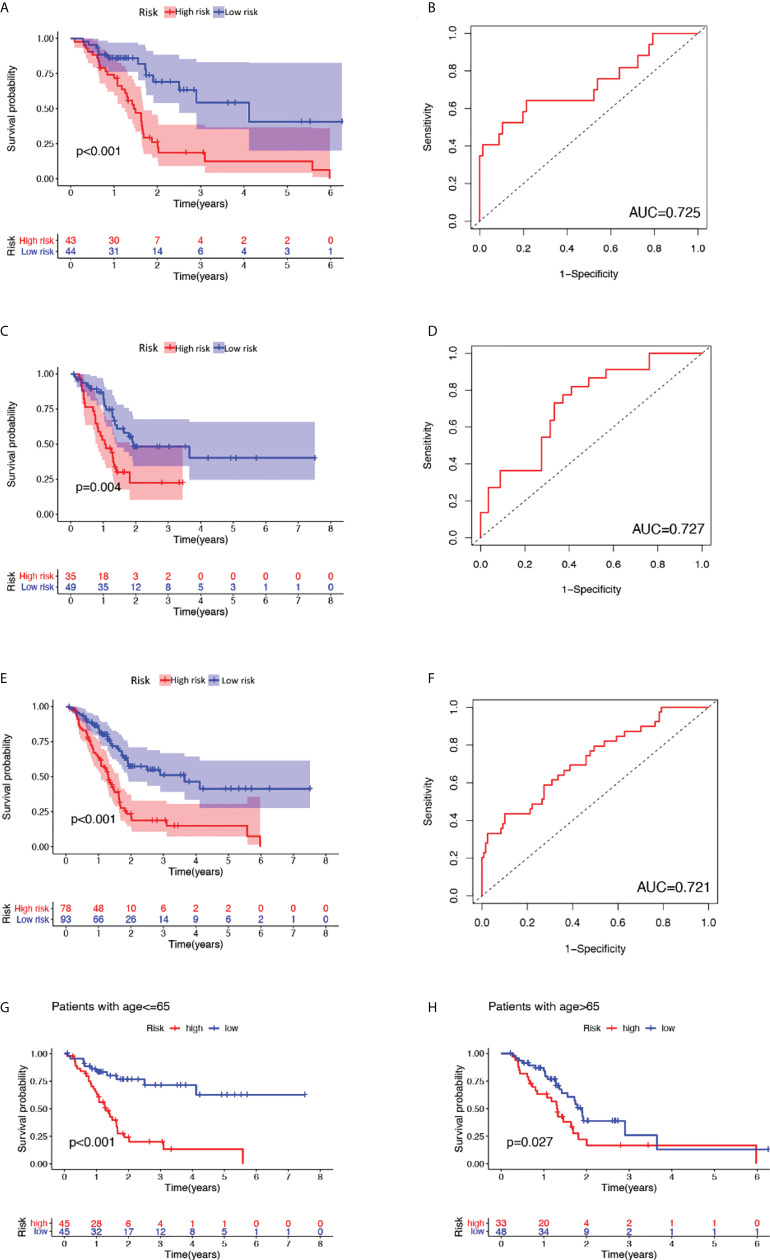
Outcome prediction of constructed GILncSig and verification as a valid prognostic factor independent of key clinical features. **(A)**, **(C)**, **(E)** Kaplan-Meier estimates of overall survival predicted by GILncSig for low-risk or high-risk patients in the training set, testing set and the combined TCGA cohort, respectively. Statistical analysis was performed using the log-rank test. **(B)**, **(D)**, **(F)** Time-dependent ROC curve analysis of GILncSig at 3 years in the training set, testing set and the combined TCGA cohort. **(G)** Kaplan-Meier estimates of overall survival predicted by GILncSig for low-risk or high-risk patients in young-patient group (age ≤ 65). **(H)** Kaplan-Meier estimates of overall survival predicted by GILncSig for low-risk or high-risk patients in old-patient group.

Afterward, a genomic instability-derived lncRNA signature was constructed based on the coefficients of the aforementioned multivariate Cox proportional hazard regression model and the expression level of siglncRNAs. The formula is as follows: GILncSig score = (0.2917 × expression level of TM4SF1-AS1) + (0.1665 × expression level of CASC8) + (-0.2782 × expression level of PRDM16-DT) + (-1.1024 × expression level of LINC00996) + (-1.0421 × expression level of AP000892.3). Of the GILncSig, the coefficient of lncRNA TM4SF1-AS1 and CASC8 were positive, suggesting that they are risk factors as their expressions were correlated with a poor prognosis, whereas the coefficient of lncRNA PRDM16-DT, LINC00996 and AP000892.3 were negative, suggesting that they are protective factors as their expressions were correlated with a better outcome. To predict the survival of pancreatic patients, the risk score for each patient in the training set was obtained through GILncSig. Using the median risk score (1.146) as the cut-off value, these patients were classified into two prognostic groups—either high-risk or low-risk groups. Kaplan-Meier analysis revealed that the survival outcomes of patients in the high-risk group are significantly worse than those in the low-risk group (median OS 1.46 years versus 4.12 years, P< 0.01, log-rank test; **Figure 2A**). The survival rate of the high-risk group was 18.6% at 3 years and that of the low-risk group was 54.3%. The area under curve (AUC) yielded by the time-dependent ROC curves analysis of GILncSig was 0.725 (**Figure 2B**). Similarly, the survival analysis and time-dependent ROC curves analysis were applied to the testing set and the combined TCGA cohort. For the testing set, Kaplan-Meier analysis revealed that the survival outcomes of patients in the high-risk group are significantly worse than those in the low-risk group (median OS 1.08 years versus 1.92 years, P = 0.04, log-rank test; **Figure 2C**). The survival rate of the high-risk group was 22.5% at 3 years and that of the low-risk group was 48.3%. The AUC yielded by the time-dependent ROC curves analysis of GILncSig was 0.727 (**Figure 2D**). For the combined TCGA cohort, Kaplan-Meier analysis revealed that the survival outcomes of patients in the high-risk group are significantly worse than those in the low-risk group (median OS 1.30 years versus 3.65 years, P< 0.01, log-rank test; **Figure 2E**). The survival rate of the high-risk group was 18.8% at 3 years and that of the low-risk group was 51.2%. The AUC yielded by the time-dependent ROC curves analysis of GILncSig was 0.721 (**Figure 2F**)

#### 3.2.3 Verification of GILncSig as a valid prognostic factor independent of key clinical features

To clarify the independence of GILncSig from other clinical features, both univariate and multivariate Cox proportional hazard regression analysis were utilized. First, we preprocessed the data of patients in TCGA and excluded data with missing grades, stages, or ages. Next, univariate Cox proportional hazard regression analysis was carried out on GILncSig score and clinical features including score, age, gender, pathological stage, and tumor grade. As a result, GILncSig score, age, and tumor grade were identified as significant prognostic factors (P< 0.05, **Table 4**). Later, multivariate Cox proportional hazard regression analysis was performed among GILncSig score, age, and tumor grade. Finally, GILncSig and age retained their prognostic significance (P< 0.05, **Table 5**), which demonstrated that GILncSig could act as an independent prognostic factor. As age is significantly correlated with the overall survival of pancreatic patients, a stratification analysis is in need to reassure that the significantly different predicted survival outcomes of the high-risk and low-risk groups determined by GILncSig were not attributed to age difference. To do so, we stratified patients in the TCGA set into a young-patient group (n = 90) and an old-patient group (n = 81) according to the median age (age = 65) of the whole TCGA cohort. Then, the GILncSig risk score for patients in each age group was calculated to further divide them into high-risk or low-risk groups. As shown in **Figure 2**, the high-risk group has significantly worse overall survival compared with the low-risk group in both the young-patient group (P< 0.001, log-rank test; **Figure 2G**) and old-patient group (P = 0.027, log-rank test; **Figure 2H**).

**Table 4:** Univariate Cox proportional hazard regression analysis revealed that GILncSig risk score, age and grade are significant prognostic factors.

Variables	Hazard ratio	P value
Age	1.02720658	0.01218871
Gender	0.87372328	0.52335853
Grade	1.39198899	0.02575897
Stage	1.36518243	0.105923
GILncSig risk score	1.01201713	0.01771823

**Table 5:** Multivariate Cox proportional hazard regression analysis using GILncSig risk score, age and grade showed that only GILncSig risk score and age retained their prognostic significance.

Variables	Hazard ratio	P value
Age	1.02728148	0.0151126
Grade	1.3172337	0.06937243
GILncSig risk score	1.01540857	0.00362892

#### 3.2.4 Alignment of GILncSig scores with somatic mutation and UBQLN4 gene expression patterns

**Figure 3:**
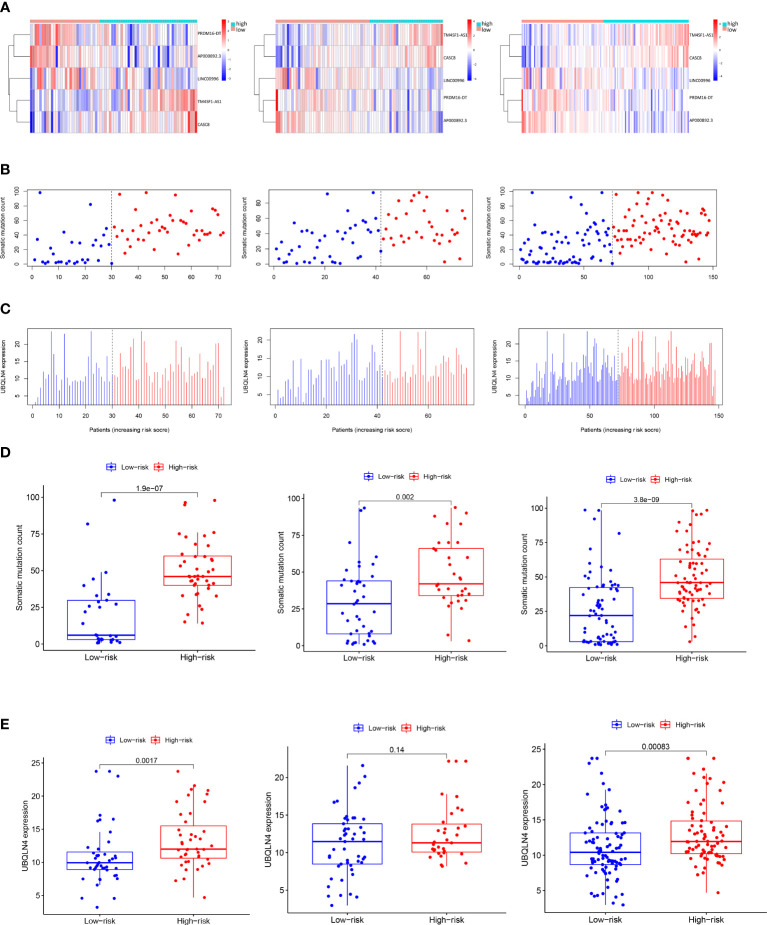
Visualization of the interconnection between GILncSig risk score and expression levels of five siglncRNAs, somatic mutation counts and expression level of UBQLN4. **(A)** Patients in the training set, testing set and the combined TCGA cohort were ranked from left to right on the X-axis according to ascending GILncSig risk score, while the expression levels of the five siglncRNAs were shown on the Y-axis. **(B)** Distribution of somatic mutation counts with increasing GILncSig score as X axis. **(C)** Distribution of UBQLN4 expression level with increasing GILncSig score as X axis. **(D)** Box plots of somatic mutations in the training set, testing set and the combined TCGA cohort in the high- and low-risk groups. Somatic mutation counts in the high-risk group were significantly higher than those in the low-risk group. **(E)** Box plots of UBQLN4 expression levels in the training set, testing set and the combined TCGA cohort in both the high-risk and low-risk groups. The UBQLN4 expression level was significantly higher in the high-risk group than in the low-risk group. Statistical analysis was performed using the Mann-Whitney U test.

We further explored the variation patterns of somatic mutation counts and UBQLN4 gene expression levels with increasing GILncSig scores to consolidate GILncSig’s association with genomic instability. As can be seen in the heatmap of **Figure 3A**, patients in the training set, testing set, and combined TCGA set were sorted from left to right based on the values of their computed GILncSig scores on the X axis, while the expression levels of 5 siglncRNAs were shown on the Y axis. The corresponding somatic mutation counts and UBQLN4 gene expression levels of these patients were acquired and plotted accordingly in **Figures 3B, C**. Upregulated expression levels of risky lncRNAs (TM4SF1-AS1, CASC8) and downregulated expression levels of protective lncRNAs (PRDM16-DT, LINC00996, AP000892.3) were detected in patients with high GILncSig scores, whereas the opposite expression patterns were detected in patients with low GILncSig scores. Statistical comparison of somatic mutation counts and UBQLN4 expression levels between high-risk and low-risk groups within the training set, testing set, and combined TCGA set was performed. It is revealed that high-risk groups have significantly higher somatic mutation counts compared with low-risk groups in all three patient sets (from left to right: P< 0.001, P = 0.002, P< 0.001, Mann–Whitney U test; **Figure 3D**) and that high-risk groups have significantly higher UBQLN4 expression levels compared with low-risk groups in the training set and combined TCGA set. Even though the difference did not reach significance in the testing set, there is still a discernible trend from the box and dot plot that the high-risk group possessed a higher expression level of UBQLN4 versus the low-risk group (from left to right: P = 0.0017, P = 0.14, P< 0.001, Mann–Whitney U test; **Figure 3E**).

### 3.3 GILncSig adds value to the current literature field of prognostic pancreatic cancer biomarker

**Figure 4:**
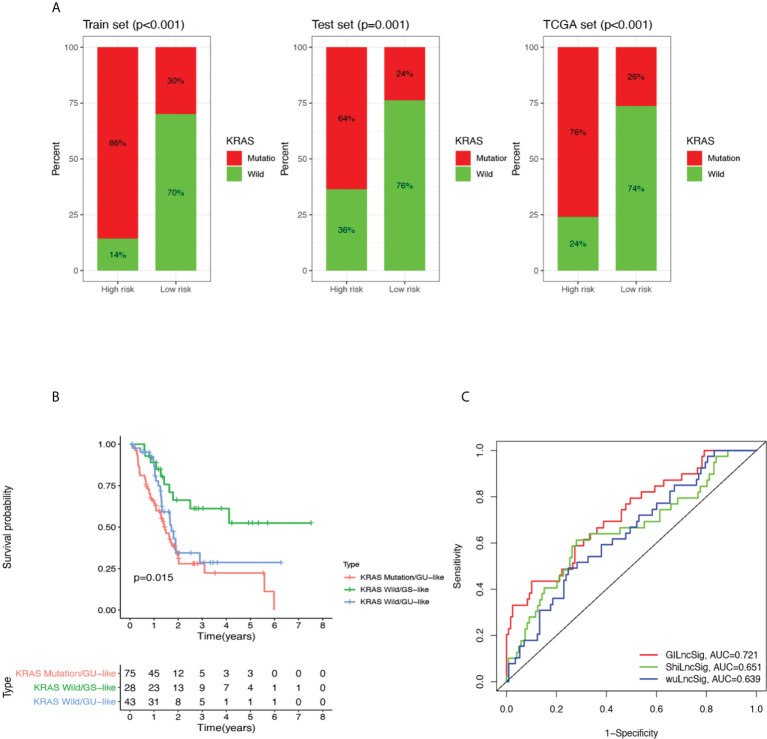
Performance comparison of GILncSig with KRAS paradigm and lncRNA-related prognostic models derived from other studies. **(A)** The proportion of KRAS mutations in high- and low-risk groups in the training set, testing set and the TCGA cohort. **(B)** Kaplan-Meier curve analysis of overall survival for PDAC patients belonging to KRAS Mutation/GU-like group, KRAS Wild/GU-like group and KRAS Wild/GS-like group for patients classified by KRAS mutation status and GILncSig. Statistical analysis was performed using the log-rank test. **(C)** The ROC analysis at 3 years of overall survival for GILncSig, WuLncSig, and ShiLncSig.

To investigate whether GILncSig can stand as a solid prognostic biomarker for pancreatic cancer, we tested its correlation with some mutated genes in pancreatic cancer and compared their survival outcome predicting capability. KRAS is a classic oncogene that is actively involved in the pathogenesis of pancreatic cancer (35–37). In addition, growing evidence revealed that KRAS is firmly implicated in the diagnosis and prognosis of pancreatic cancer and is heralded as a potential therapeutic target (35, 37). First of all, we compared the proportion of patients with KRAS mutation within the high-risk and low-risk groups, and as can be seen in **Figure 4A**, high-risk groups occupied a higher percentage of patients with KRAS mutation compared with low-risk groups in the training set, testing set, and combined TCGA set. Next, we categorized patients from the TCGA set into four different sub-groups based on their KRAS mutation status and GILncSig risk group membership. In other words, the following four groups were classified: KRAS Mutation/GU-like group, KRAS Mutation/GS-like group, KRAS Wild/GU-like group and KRAS Wild/GS-like group. Since there was only 1 patient who belonged to the KRAS Mutation/GS-like group, this group was removed from the following analysis. Later, survival analysis was performed. As can be inferred from **Figure 4B**, the survival outcome was significantly different among these three groups (P = 0.015). KRAS Mutation/GU-like group was predicted to have the worst outcome (median survival time: 1.46 years, 3-year survival rate: 28.0%), KRAS Wild/GS-like group was predicted to have the best outcome (3-year survival rate: 61.2%), whereas KRAS Wild/GU-like group was in between (median survival time: 1.72 years, 3-year survival rate: 28.7%). Our data indicated that GILncSig is able to identify a sub-population of pancreatic cancer patients who might be at a higher mortality rate and thus deserve a more radical treatment regimen that could otherwise go unnoticed due to their KRAS wild type status. Results for other pancreatic cancer-associated mutated genes were also consistent with KRAS, and results for TP53 are presented in **Supplementary Figure 3**. Therefore, we believe GILncSig can be an asset to the current literature field of prognostic pancreatic cancer biomarkers.

### 3.4 Performance comparison of GILncSig with existing lncRNA-related signatures in survival prediction

Finally, we compared the prediction performance of GILncSig with two recently published lncRNA signatures: 3-lncRNA signature obtained from Wu’s study (hereinafter referred as WuLncSig) (38) and 3-lncRNA signature derived from Shi’s study (hereinafter referred as ShiLncSig) (39) using our TCGA patient cohort. As shown in **Figure 4C**, the AUC at 3 years of OS for our GILncSig is 0.721, which is significantly higher than WuLncSig (AUC = 0.639) and significantly higher than ShiLncSig as well (AUC = 0.651). Even though both WuLncSig and ShiLncSig used a smaller number of lncRNAs (n = 3) than GILncSig (n = 5), we still maintained that our signature should be considered the better model since it can offer a more accurate prediction.

### 3.5 Functional regulation of GILncSig in pancreatic cancer is potentially associated with EMT and lack of adaptive immunity participation within the TME

#### 3.5.1 Clustering analysis of TCGA dataset yields three different clusters of pancreatic cancer patients

**Figure 5:**
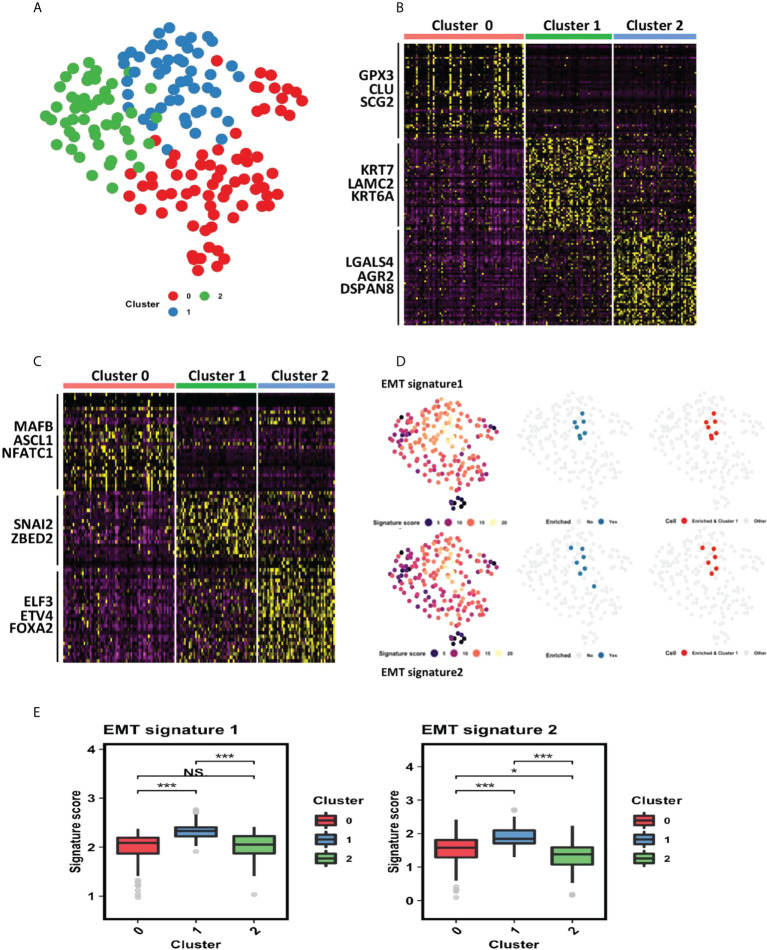
Cluster 1 identified by the clustering analysis of PDAC TCGA dataset is associated with epithelial–mesenchymal transition (EMT). **(A)** Clustering analysis of PDAC TCGA dataset identified 3 distinct clusters (cluster 0, cluster 1 and cluster 2) projected onto UMAP coordinates. **(B)** Heatmap showing top 50 differentially expressed genes for each cluster. Red represents cluster 0, green represents cluster 1, and blue represents cluster 2. **(C)** Heatmap showing top 30 differentially expressed transcription factors for each cluster. Red represents cluster 0, green represents cluster 1, and blue represents cluster 2. **(D)** Gene set enrichment analysis in all three clusters of PDAC patients. The tested gene sets were gene signatures extracted from previous literature that are strongly related to EMT. The first column showcases the signature score each patient earns. The higher the signature score, the more enriched the corresponding patient is for each gene set. The second column showcases all patients with a high signature score (ranked at 95th percentile or above). The third column showcases that patients highly enriched for each gene set all belong to cluster 1. **(E)** Statistical comparison of EMT signature scores of patients in the three clusters. *P< 0.05; ***P< 0.001; Ns, not significant.

To further understand the potential functional regulatory effects of GILncSig, an explorational graph-based clustering analysis was performed for the whole TCGA dataset. As a result, three distinct clusters were identified among all pancreatic cancer patients and projected onto the UMAP coordinate shown in **Figure 5A**. The clusters were labeled as cluster 0, cluster 1, and cluster 2 respectively. Top 50 differentially expressed genes and top 30 differentially expressed transcription factors for each cluster were extracted and shown in the heatmaps (**Figures 5B, C**).

#### 3.5.2 Cluster 1 is associated with EMT

Via close examination of the differentially expressed genes and transcription factors derived from each cluster, it can be inferred that cluster 1 is related to EMT, as SNAI2 and ZBED2, which are pivotal EMT-inducing transcription factors, are differentially upregulated within cluster 1 (40, 41). To further confirm this association, we carried out a gene set enrichment analysis using two EMT signatures from previous literature (32, 42). As shown in **Figure 5D**, patients’ enriched-ness in expression of genes within each gene set were defined by a signature score calculated through R VISION package. We observed that patients highly enriched for each gene set (whose signature score is ranked at 95th percentile or above) all belonged to cluster 1, further statistical comparison also consolidated that those patients of cluster 1 have the highest EMT signature score (**Figure 5E**).

#### 3.5.3 GILncSig is associated with EMT

**Figure 6:**
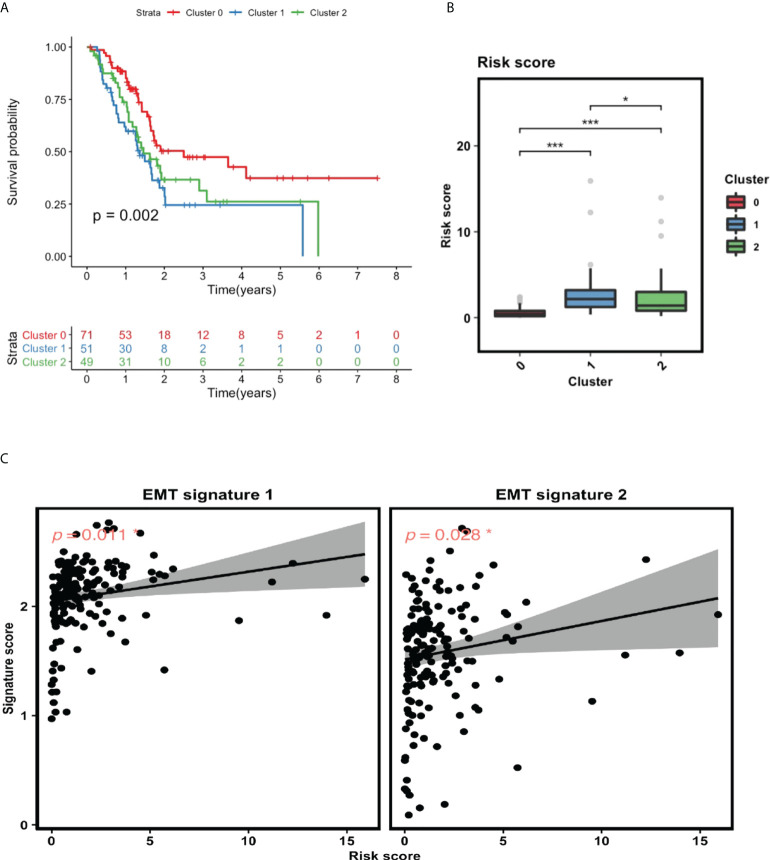
GILncSig is tightly linked to EMT. **(A)** Survival analysis for patients in three clusters. **(B)** Statistical comparison of GILncSig risk scores for the patients in three clusters. **(C)** Pearson correlation analysis between GILncSig risk score and EMT signature score. *P< 0.05; ***P< 0.001.

To explore the relationship between GILncSig and the identified clusters, we performed survival analysis for these three clusters and compared their GILncSig risk score levels. As shown in **Figure 6A**, patients from cluster 1 have the worst prognosis, whereas patients from cluster 0 have the best prognosis. Interestingly, the survival outcome of the patients from these three clusters corresponds tightly to their GILncSig risk score levels, with patients belonging to cluster 1 having the highest GILncSig risk score and patients belonging to cluster 0 harboring the lowest GILncSig risk score (**Figure 6B**). Furthermore, Pearson correlation analysis showed that the GILncSig risk score is significantly positively associated with EMT signature scores (**Figure 6C**). Together, these data indicate that pancreatic cancer patients with high GILncSig risk score are more likely to undergo EMT within the tumor, which in return may confer them a worse survival outcome.

#### 3.5.4 TME estimation revealed inadequacy of adaptive immunity participation within GILncSig high-risk group

**Figure 7:**
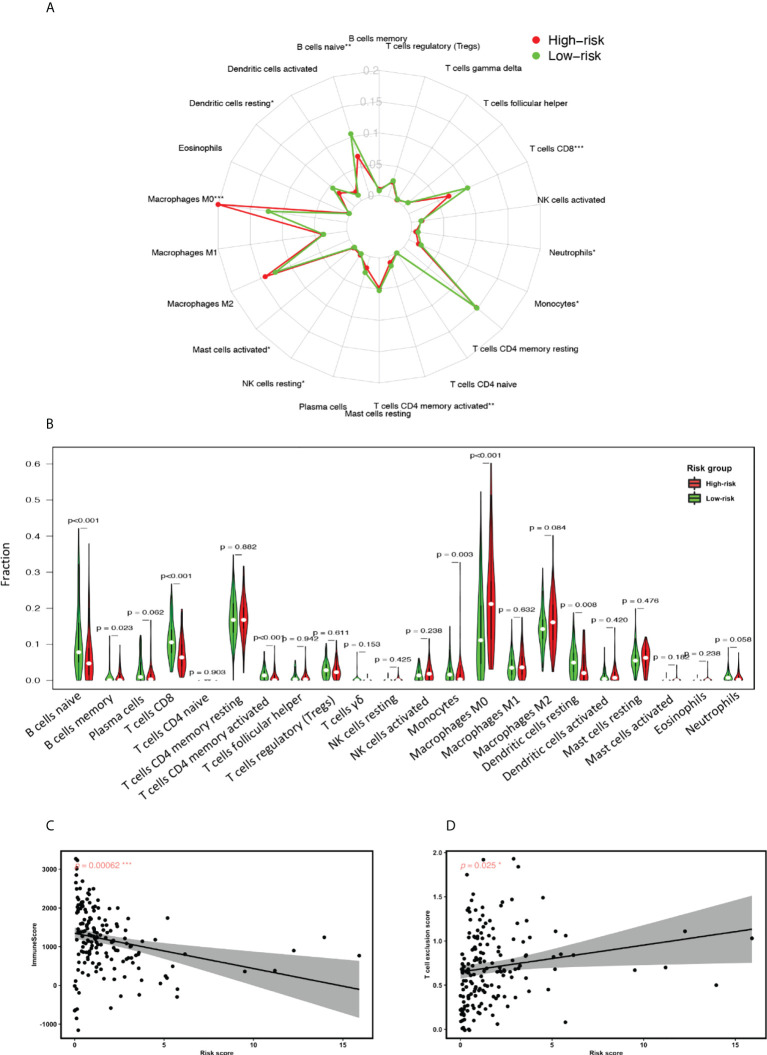
GILncSig associated tumor microenvironment (TME) assessment. **(A)** Radial plot highlighting the differences of the median proportion of 22 immune cell types in the TME of PDAC patients in the high-risk and low-risk groups. **(B)** Statistical comparison of the fraction of 22 immune cell types in the TME of patients in the high-risk and low-risk groups assigned based on GILncSig. Red represents the high-risk group and green represents the low-risk group. **(C)** Pearson correlation analysis between Immune Score and GILncSig risk score. **(D)** Pearson correlation analysis between T cell exclusion score and GILncSig risk score. *P< 0.05; **P< 0.01; ***P< 0.001.

Finally, we conducted TME estimation using the CIBERSORT algorithm to understand the GILncSig-related immune landscape. Statistical comparison of the concentration of 22 immune cell types within the TME revealed a strikingly diminished adaptive immunity participation in the GILncSig high-risk group. As can be seen from **Figures 7A, B**, the concentration of naive B cells, activated CD4+ memory T cells and CD8+ T cells were significantly reduced in the GILncSig high-risk group. This observation is further affirmed by the Pearson correlation analysis, which showed that the ImmuneScore is significantly inversely correlated with GILncSig risk score, while T cell exclusion score is significantly positively correlated with GILncSig risk score (**Figures 7C, D**). Together, these data suggest that the worse survival outcome of pancreatic cancer patients from the GILncSig high-risk group might be in part attributed to the inadequacy of robust adaptive immune cells, i.e., B cell and T cell infiltration within the TME.

In summary, our data provide evidence that the aberrant expression pattern of GILncSig in pancreatic cancer patients can lead to a more invasive cancer subtype, thereafter rendering them a worse survival prognosis. This may be achieved through the regulation of genes promoting EMT and the hindrance of adaptive immune cell infiltration within the TME.

### 3.6 Validation of lncRNA signature by real-time quantitative PCR

**Figure 8:**
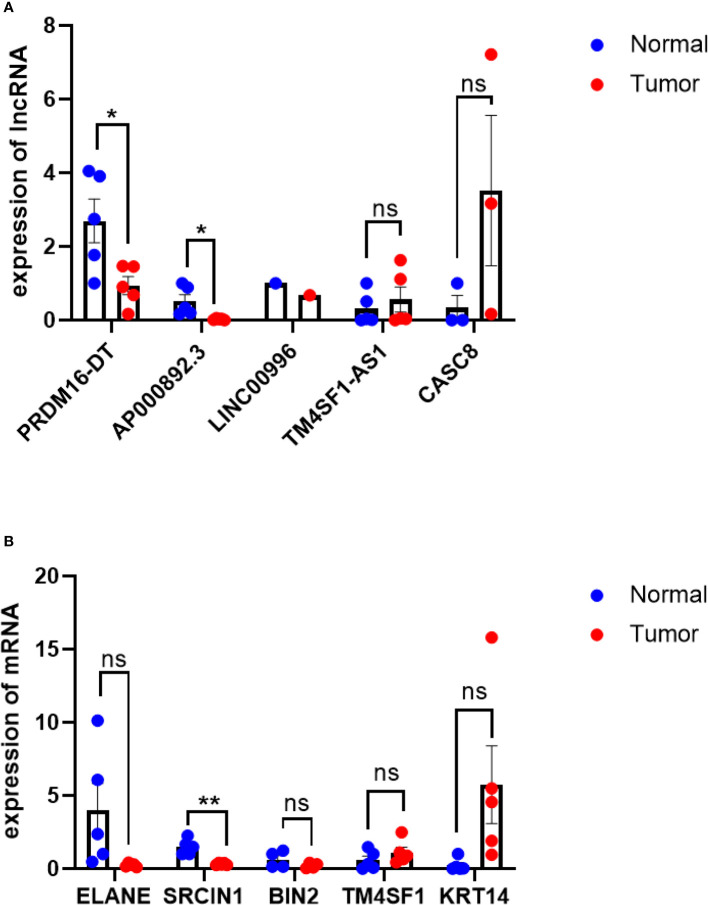
qRT-PCR of lncRNAs and their target genes. **(A)** qRT-PCR results of lncRNAs, PDAC tissue VS normal tissue. **(B)** qRT-PCR results of target genes,KRT14 is highly expressed in PDAC tissues(p=0.07), ELANE expression is lower in normal tissues(P=0.07), PDAC tissue VS normal tissue. *P< 0.05, **P< 0.01; Ns, not significant.

The expression levels of all lncRNAs and their target genes in five pancreatic ductal adenocarcinoma tissues and matched normal tissues were detected using qRT-PCR. Compared with adjacent normal pancreas, the mRNA expression of TM4SF1-AS1 and CASC8 was higher, while the expression of PRDM16-DT(P<0.05), AP000892.3(P<0.05), and LINC00996 was lower in PDAC tissues (**Figure 8A**). The expression trend of target genes was consistent with that of lncRNAs (**Figure 8B**). The results of our validation were consistent with the model, but the statistics of most of the lncRNAs did not show significance due to the large difference in cancer tissues among different individuals.

## 4 Discussion

Here, we established a computational framework for the identification of genomic instability-related lncRNAs, through which 206 lncRNAs associated with genomic instability were identified, and five lncRNAs (TM4SF1-AS1, CASC8, PRDM16-DT, LINC00996, AP000892.3) were selected from them as independent prognostic factors for constructing GILncSig. It has been found that mutations in key genes or aberrant signaling pathways drive the pathogenesis of PDAC, such as the mutation of oncogene KRAS and the frequent inactivation of tumor suppressors including TP53, SMAD4, and CDKN2A. Moreover, these gene mutations converge in KRAS, TGF-β, Wnt, Notch, and ROBO/SLIT signaling pathways as well as chromatin remodeling, DNA repair and other pathways and processes (43). This suggests that the high heterogeneity of PDAC is achieved by the overactivation of many signaling pathways related to growth and proliferation and the alteration of the expression levels of tumor suppressor genes, thereby affecting cell proliferation, survival and invasion. KRAS mutations, on the other hand, are considered the earliest event in PDAC initiation (44). Therefore, we compared the predictive ability of GILncSig with KRAS for survival outcome and found that GILncSig was able to identify pancreatic cancer patients with KRAS mutations who may have a higher mortality rate than the rest of the patients. Studies have pointed out that the different subtypes defined by gene expression patterns and clinical features of patients with pancreatic cancer may be of great value in predicting the prognosis of patients and guiding precision medicine (45).

In addition, three of the five selected lncRNAs associated with genomic instability (PRDM16-DT, LINC00996, AP000892.3) were protective factors, while TM4SF1-AS1 and CASC8 often served as risk factors associated with poor prognosis (46–50). These lncRNAs have been demonstrated to play an important role in the occurrence, development, and prognosis of a variety of malignant tumors (51–53). However, most of them were found to be associated with the prognosis of PDAC for the first time. Notably, CASC8 is not only strongly associated with poor survival in pancreatic ductal adenocarcinoma, but may also be involved in the process of EMT by competitively binding miR-671 (54).

EMT is a complex biological trans-differentiation process that allows epithelial cells to transiently acquire mesenchymal features, including motility and metastatic potential (55, 56). Activation of EMT is thought to be a major driver of tumor progression from initiation to metastasis. For instance, the EMT transcription factor Zeb1 is not only a key factor in lesion formation, invasion and significant metastasis, but also can affect the stemness and colonization ability of tumor cells, especially phenotypic/metabolic plasticity (55). Further studies revealed that lncRNA Linc-ROR can promote tumor invasion and metastasis by regulating Zeb1 (57). Besides, Zhu, W. et al. found that overexpression of lncRNA-CASC8 resulted in up-regulation of TOB1 and low expression of miR-129-5p, which were associated with an increased frequency of lymph node metastasis and a higher trend of pathological stage, respectively, thus validating the CASC8-miR-129-5p-TOB1 regulatory axis (58). These studies all suggested the association of GILncSig with EMT, which we also confirmed in the present study.

The tumor microenvironment of PDAC contains immune components such as interstitial cells, inflammatory cells, and cytokines, which comprise a complex network to promote tumor growth and invasion. It has also been shown that the TME of PDAC is closely related to EMT as well as KRAS (24, 59). So, we used CIBERSORT and estimation algorithms to analyze the details of GILncSig-associated TME profiles, including the estimated proportion of tumor-infiltrating immune cells (TICs) and the quantification of adaptive immune cell exclusion level. The results showed that naïve B cells, activated CD4 + memory T cells, and CD8 + T cell concentrations were significantly lower in the high-risk group. The immune score and T cell exclusion score were significantly negatively and positively correlated with the GILncSigrisk score, respectively, suggesting that this model can predict the degree of infiltration of immune cells. It has been found that reduced infiltration of these adaptive immune cells is associated with the development of pancreatic cancer armed with immune evasion mechanisms and may also impair the effect of immunotherapy in patients (60–63). Therefore, we believe that the treatment of patients can be better guided with the help of this model, especially in the responsiveness of immunotherapy. To our knowledge, this is the first and most comprehensive study to date describing the prognostic and immunotherapeutic response predictive value of TME in patients with PDAC.

Although our study provides important insights for evaluating genomic instability and the prognosis of patients with pancreatic ductal adenocarcinoma, and reveals the association of GILncSig with PDAC immune profiles, there are still some limitations. First, the TCGA cohort containing 171 patients was relatively smaller than the cohort of patients with other cancer types such as breast or lung cancer. In addition, GILncSig was validated only in the TCGA dataset and in five patient specimens due to the lack of a reliable large independent dataset. These shortcomings can only be remedied by further development of these public databases in the future. Second, we need further functional studies to understand the exact regulatory mechanism of GILncSig in maintaining genomic instability. Similarly, the GILncSig-associated tumor microenvironment is achieved by bioinformatics methods, so the results may require more in-depth studies to confirm. Third, there is no data on immunotherapy in the TCGA dataset, so the predictive power of GILncSig for the responsiveness to immunotherapy is indirectly assessed.

In conclusion, we identified lncRNAs associated with genomic instability through a computational framework based on the mutant hypothesis in the present study. By combining lncRNA expression profiles, somatic mutation profiles, and clinical information of pancreatic cancers as case studies, we identified a genomic instability-derived lncRNA signature as an independent prognostic marker to stratify pancreatic cancer patients at risk and validated it in the TCGA cohort. In addition, we used CIBERSORT and estimation algorithms to comprehensively understand the tumor microenvironment in pancreatic cancer patients. Lastly, through the functional enrichment analysis of a distinct cluster of PDAC patients with high GILncSig scores and worse survival prognosis, we found that the distraught expression of genes that promote EMT and prevent adaptive immune cell infiltration in the TME may be the key down-stream regulatory network for GILncSig.

## Data availability statement

The datasets presented in this study can be found in online repositories. The names of the repository/repositories and accession number(s) can be found in the article/**Supplementary Material**.

## Ethics statement

This study was approved by the local Ethics Committee (Second Xiangya Hospital Ethics Committee) (approved no. 2020-465). Written informed consent for participation was not required for this study in accordance with the national legislation and the institutional requirements.

## Author contributions

HY and WZ conceived the study and wrote the manuscript. LX and JH collected the information of patients with pancreatic ductal adenocarcinoma from public databases. JD and WZ performed statistical analysis as well as functional enrichment analysis of the data. YS and HY collected tissue specimens from PDAC patients and completed the validation of GILncSig. ZM, ZS, WP, and YC modifified the methods, expressions, and contents in the study, respectively. YX and ZS are responsible for all aspects of the work to ensure that questions related to the accuracy or integrity of any part of the work are appropriately investigated and resolved. All authors contributed to the article and approved the submitted version.

## Funding

This work was supported by the National Key Research and Development Program (2018YFE0114500 to YX), the National Science Foundation of Hunan Province for Excellent Young Scholars (2020JJ3056 to YX), the National Natural Science. Foundation of China (NSFC) grant (81870577 to YX), the Natural Science Foundation of Hunan Province for Youths (2022JJ40718 to LX), the Natural Science Foundation of Changsha (kq2202404 to JH), and the Natural Science Foundation of Hunan Province for Youths (2022JJ40689 to JH).

## Conflict of interest

The authors declare that the research was conducted in the absence of any commercial or financial relationships that could be construed as a potential conflict of interest.

## Publisher’s note

All claims expressed in this article are solely those of the authors and do not necessarily represent those of their affiliated organizations, or those of the publisher, the editors and the reviewers. Any product that may be evaluated in this article, or claim that may be made by its manufacturer, is not guaranteed or endorsed by the publisher.
