# Supplementary material for: A novel genomic instability-derived lncRNA signature to predict prognosis and immune characteristics of pancreatic ductal adenocarcinoma
Source: Front Immunol. 2022 Sep 15;13:970588. doi: 10.3389/fimmu.2022.970588 (PMC9486402; doi:10.3389/fimmu.2022.970588)
Supplement: Supplementary file 1 [file DataSheet_1.docx]

## 1 Supplementary Figures


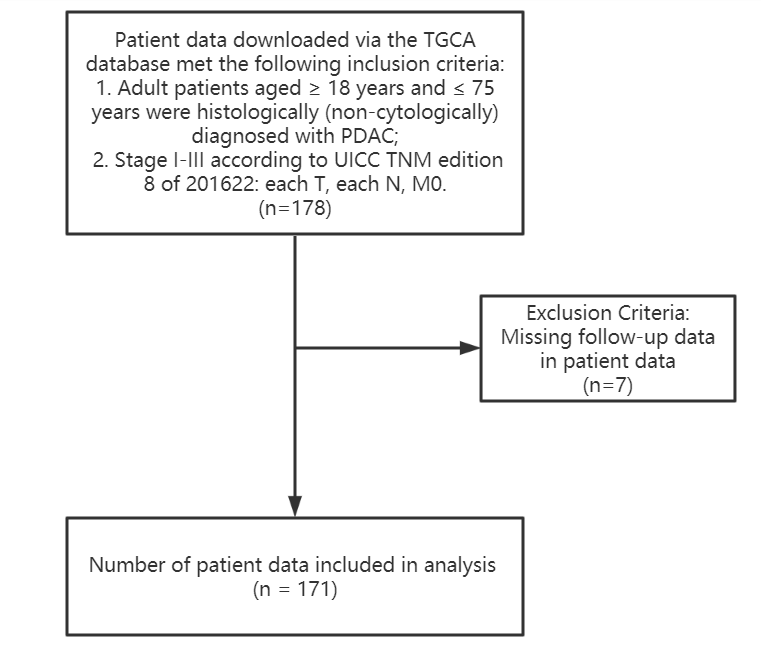


**Supplementary Figure 1.** The flowchart of the inclusion and exclusion criteria for patient data in the TCGA cohort.


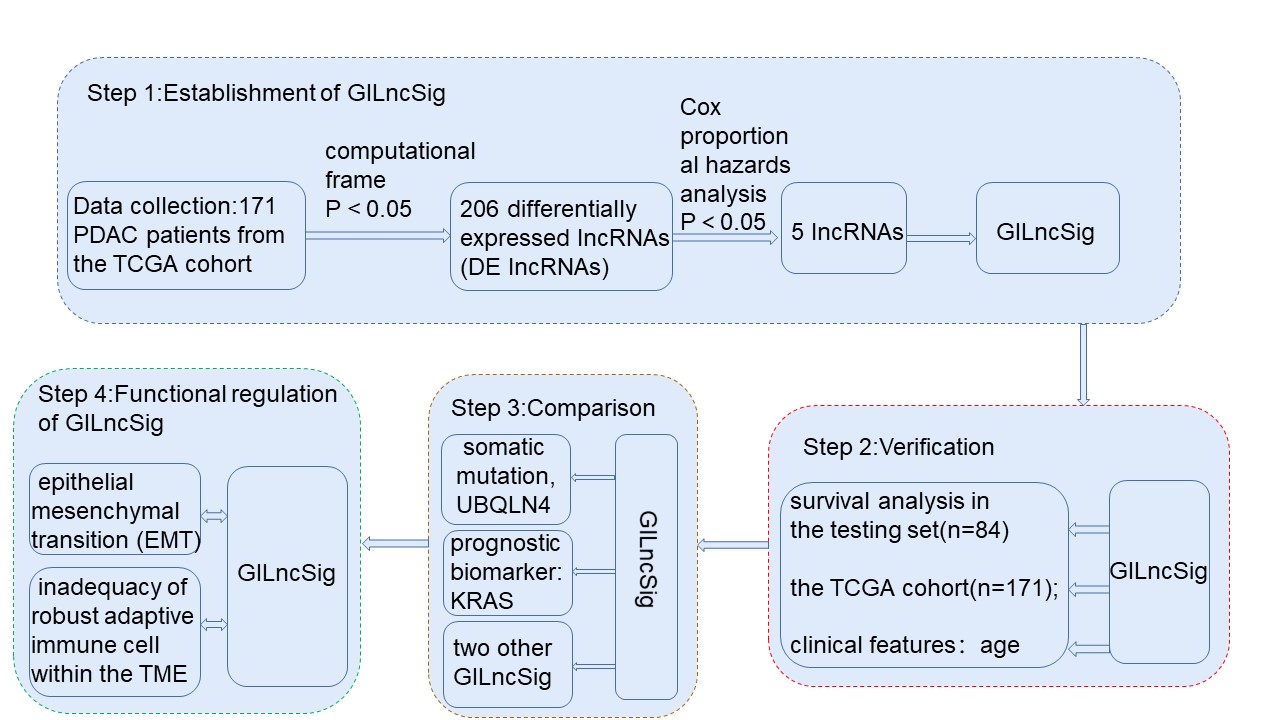


**Supplementary Figure 2.** The flowchart of this study. 171 PDAC patients from the TCGA cohort were recruited in this study. The computational frame is a mutator hypothesis-derived computational frame combining lncRNA expression profiles and somatic mutation profiles in a tumor genome was used to detect genome-instability related lncRNAs. Cox proportional hazards analysis was performed on the genome-instability related lncRNAs and key patient clinical features to screen for lncRNAs independently associated with patient survival. These lncRNAs were then incorporated to build GILncSig. Survival analysis was conducted to verify the prognostic value of GILncSig. Stratefication analysis using clinical feature of age was carried out to clarify the independent prognostic capacity of GILncSig. Somatic mutation burden and expression level of UBQLN4 were compared between high-risk and low-risk groups assigned using GILncSig to confirm the association between GILncSig and genome instability. Survival analysis is performed on groups of PDAC patients with different combination of GILncSig risk group status and KRAS mutation status to assess if the risk predicting capability of GILncSig is on par with that of KRAS, a classic mutated oncogene in PDAC patients heralding significantly increased risk. To declare that our GILncSig is so far the best genomic-instability associated lncRNAs derived prognostic model for PDAC, The ROC analysis was performed using our GILncSig and two other GILncSigs established by other study groups (WuLncSig and ShiLncSig^).^ Finally, functional regulation of GILncSig was explored, revealing that GILncSigs was closely linked to EMT and adaptive immunity deficient TME profile.


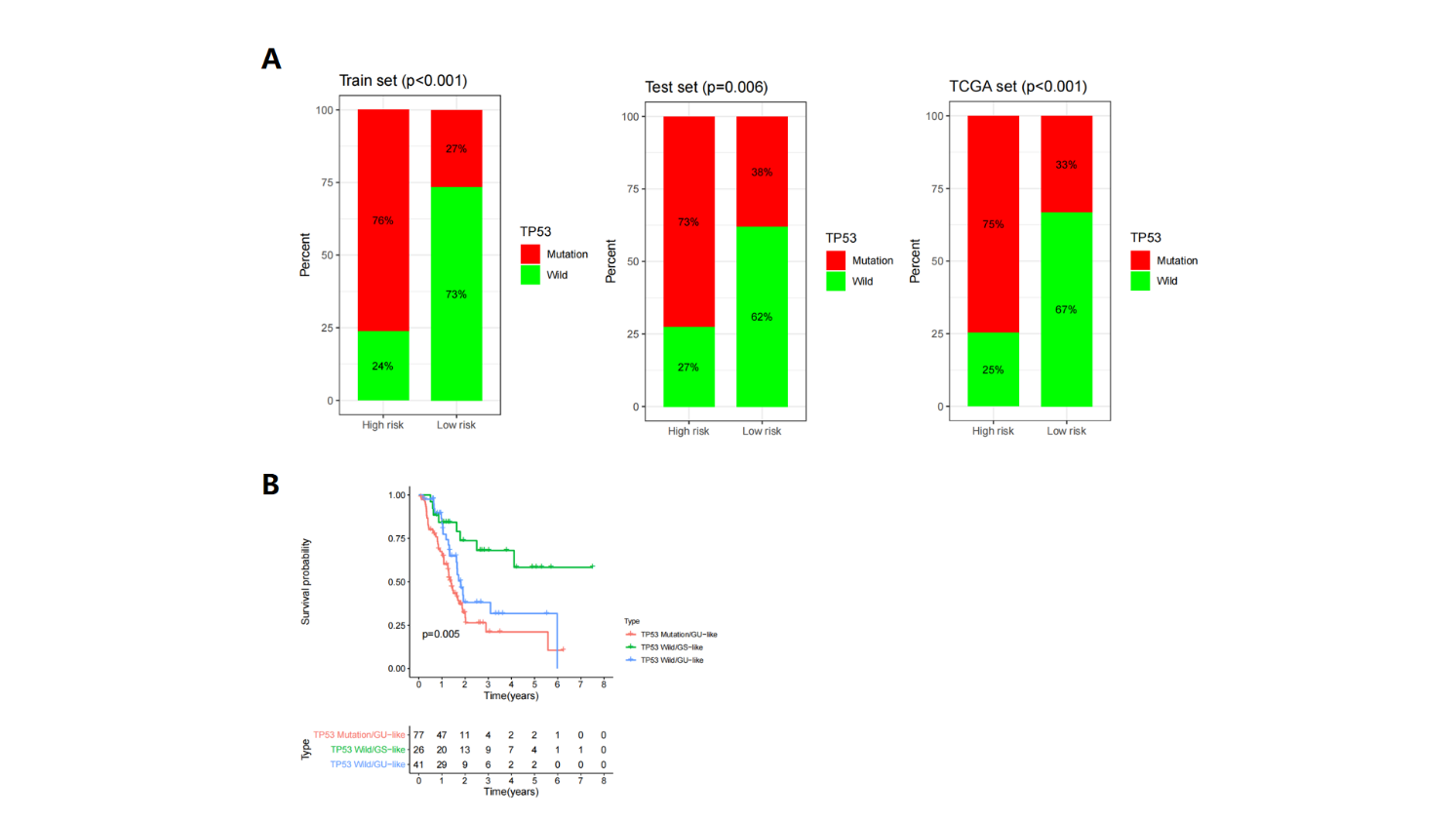


**Supplementary Figure 3.** (A) The proportion of TP53 mutations in high- and low-risk groups in the training set, testing set and the TCGA cohort. (B) Kaplan-Meier curve analysis of overall survival for PDAC patients belonging to TP53 Mutation/GU-like group, TP53 Wild/GU-like group and TP53 Wild/GS-like group for patients classified by TP53 mutation status and GILncSig. Statistical analysis was performed using the log-rank test.

## 2 Supplementary Tables

**Supplementary Table 1 The primer sequences used in this study**

| **Gene** | **Primer** | **Sequence (5′-3′)** |
| --- | --- | --- |
| TM4SF1-AS1 | forward | ACAACAGAGGTGGCATCAGTCAAC |
|  | reverse | TTGTCCTTGTGGCATCTTCAACTCC |
| TM4SF1 | forward | GGCTACTGTGTCATTGTGGCAG |
|  | reverse | ACTCGGACCATGTGGAGGTATC |
| CASC8 | forward | ACATAACAGCCACCCAAAA |
|  | reverse | CCACATGAAGCAGACACAA |
| KRT14 | forward | GAACCACGAGGAGGAGATGAATGC |
|  | reverse | CGTTCAGAATGCGGCTCAGGTC |
| PRDM16-DT | forward | AGGACGTAGGTCTTTTGGCACC |
|  | reverse | CACCTAGAATGGTAGTTTGTGCC |
| ELANE | forward | AACGGGCTAATCCACGGAATTGC |
|  | reverse | TCCTCGGAGCGTTGGATGATAGAG |
| AP000892.3 | forward | ACAGGGGAAGTGTTCCTTTGG |
|  | reverse | CACCACCTTTATTATCCCTCACG |
| SRCIN1 | forward | TGCGCTATCTCAACGACGAGGA |
|  | reverse | TTCAGCACCAGTGCCTTCTCCT |
| LINC00996 | forward | GAGGGCACTTTGTCTTACTTGGC |
|  | reverse | ATTCTTCATGCCAATCCTCTCAC |
| BIN2 | forward | GGCTGCTATGTGACCATCTTCC |
|  | reverse | GCTTCTCCAGTTTGCTCATCACC |
| GAPDH | forward | GTCTCCTCTGACTTCAACAGCG |
|  | reverse | ACCACCCTGTTGCTGTAGCCAA |
